# The dynamics of the microbiome in Ixodidae are shaped by tick ontogeny and pathogens in Sarawak, Malaysian Borneo

**DOI:** 10.1099/mgen.0.000954

**Published:** 2023-02-09

**Authors:** Alice C. C. Lau, Wessam Mohamed Ahmed Mohamed, Ryo Nakao, Manabu Onuma, Yongjin Qiu, Nobuyoshi Nakajima, Michito Shimozuru, Jayasilan Mohd-Azlan, Mohamed Abdallah Mohamed Moustafa, Toshio Tsubota

**Affiliations:** ^1^​ Laboratory of Wildlife Biology and Medicine, Department of Environmental Veterinary Sciences, Faculty of Veterinary Medicine, Hokkaido University, Sapporo, 060-0818, Japan; ^2^​ Laboratory of Parasitology, Department of Disease Control, Faculty of Veterinary Medicine, Hokkaido University, Sapporo, 060-0818, Japan; ^3^​ Division of Bioinformatics, International Institute for Zoonosis Control, Hokkaido University, Sapporo, Hokkaido, 001-0020, Japan; ^4^​ Department of Biochemistry and Microbiology, Rutgers The State University of New Jersey, New Brunswick, 08901, New Jersey, USA; ^5^​ Biodiversity Division, National Institute of Environmental Studies, Tsukuba, 305-806, Japan; ^6^​ Division of International Research Promotion, International Institute for Zoonosis Control, Hokkaido University, Sapporo, 001-0020, Japan; ^7^​ Institute of Biodiversity and Environmental Conservation, Universiti Malaysia Sarawak, Kota Samarahan, 94300, Sarawak, Malaysia; ^8^​ Department of Animal Medicine, Faculty of Veterinary Medicine, South Valley University, Qena, 83523, Egypt; ^9^​ Department of Microbiology, Biochemistry and Molecular Genetics, Rutgers New Jersey Medical School, Newark, New Jersey, USA

**Keywords:** microbiome, next-generation sequencing, pathogens, Sarawak, ticks

## Abstract

Tick-borne diseases have recently been considered a potential emerging public health threat in Malaysia; however, fundamental studies into tick-borne pathogens and microbiome appear limited. In this study, six tick species (*Ixodes granulatus, Haemaphysalis hystricis, Haemaphysalis shimoga*, *Dermacentor compactus*, *Dermacentor steini* and *Dermacentor atrosignatus*) collected from two primary forests and an oil palm plantation in Sarawak, Malaysian Borneo, were used for microbiome analysis targeting bacterial 16S rDNA using next-generation sequencing (NGS). In addition, bacterial species were further characterized in conventional PCRs to identify potential pathogens. Sequences generated from NGS were first filtered with the Decontam package in R before subsequent microbial diversity analyses. Alpha and beta analyses revealed that the genus *Dermacentor* had the highest microbial diversity, and *H. shimoga* significantly differed in microbial composition from other tick species. Alpha and beta diversities were also significantly different between developmental stages of *H. shimoga*. Furthermore, we observed that some bacterial groups were significantly more abundant in certain tick species and developmental stages of *H. shimoga*. We tested the relative abundances using pairwise linear discriminant analysis effect size (LEfSe), which also revealed significant microbial composition differences between *Borrelia *-positive and *Borrelia *-negative *I. granulatus* ticks. Finally, pathogenic and potentially pathogenic bacteria circulating in different tick species, such as *Rickettsia heilongjiangensis *, *Ehrlichia* sp., *Anaplasma* sp. and *Bartonella* spp. were characterized by PCR and sequencing. Moreover, *Coxiella* and *Francisella *-like potential symbionts were identified from *H. shimoga* and *D. steini*, respectively. More studies are required to unravel the factors associated with the variations observed in this study.

## Data Summary

Ticks (16S rDNA: LC602422–LC602456 and LC603786); *Anaplasmatacea* (16S rDNA: LC602250–LC602251); *Francisella* (16S rDNA: LC602252–LC602256; *tul4*: LC602776–LC602781); *Rickettsia* (16S rDNA: LC602357–LC602360; *ompA*: LC602733–LC602736; *ompB*: LC602737–LC602740; *gltA*: LC602741–LC602744; *Sca4*: LC602745–LC602748; *htrA*: LC602770–LC602773); *Bartonella* (*ftsZ*: LC602774; *gltA*: LC602775); *Coxiella* (23S rDNA: LC602368–LC602388; 16S rDNA: LC602389–LC602400; *dnaK*: LC602703–LC602712; *rpoB*: LC602713–LC602732; *groEL*: LC602749–LC602769).

The authors confirm all supporting data, code and protocols have been provided within the article or through supplementary data files.

**Impact Statement**

In this study, we collected as many tick samples as possible by sampling in two primary forests and an oil palm plantation in Sarawak. These tick samples, after species identification, were examined by NGS to survey microbiomes and pathogens by targeting bacterial 16S rDNA. We described the microbial variations from six tick species of three genera: *Ixodes, Haemaphysalis* and *Dermacentor*. In addition, we identified the potential symbionts in most tick species and each developmental stage of *Haemaphysalis shimoga,* which could be the symbionts of respective tick species and stages. We found a significant microbial difference between different tick species, and there was ontogeny, blood meal and pathogen influence on microbial composition in ticks. Bacteria species of the genera *Anaplasma, Ehrlichia, Bartonella*, *Coxiella, Francisella* and *Rickettsia* were characterized in this study, in which *Rickettsia heilongjiangensis *, *Anaplasma* sp. closely related to *A. platys *, and *Ehrlichia* sp. closely related to *E. ewingii* were human pathogens identified in this study. This study is the first initiative to outline the tick microbiome and pathogen screening from this region and provides important insights that will lead to future studies.

## Introduction

In the tropics such as Malaysia, ticks are the second most important vector after mosquitoes causing human vector-borne diseases [1]. To date, at least 45 tick species have been documented in Malaysia [2]; amongst these, tick species belonging to the genera *Ixodes*, *Haemaphysalis*, *Dermacentor* and *Amblyomma* have been frequently documented from the vegetation and wildlife of primary, fringe and secondary forest habitats or livestock farms in Malaysia [3]. Moreover, ticks are competent vectors that harbour and transmit zoonotic pathogens, which pose significant public health threats. For instance, ticks harbour medically important bacterial species from a broad range of genera, including *Anaplasma *, *Borrelia *, *Bartonella *, *Coxiella *, *Ehrlichia* and *Rickettsia *. Some of them cause prevalent diseases, such as Lyme disease (e.g. *Borrelia afzelii *), human granulocytic anaplasmosis (* Anaplasma phagocytophilum *), and spotted fever rickettsiosis (e.g. *Rickettsia rickettsii *). However, tick-bite human cases are rarely reported in Malaysia [4], which could be due to a general lack of awareness of tick-borne diseases (TBDs). TBDs are generally poorly studied in Malaysia, as early reports in humans focused mainly on scrub typhus, a rickettsial disease transmitted by mites [5]. In more recent years, there has been an increase in studies of TBDs relating to seroprevalence screening in humans and tick surveillance.

In Malaysia, serological evidence for *Borrelia* [6, 7], *Anaplasma *, *Ehrlichia* and *Rickettsia* spp*.* [8–10] infections have been detected from certain communities such as farmworkers and indigenous people residing in Peninsular Malaysia. Nonetheless, surveillance has been limited to specific populations, and the actual bacterial strains causing these infections were mostly unknown. In addition, studies have previously detected the aetiological agents for Lyme disease, spotted fever rickettsiosis and Q fever from several tick species in Malaysia. For instance, *Borrelia* species belonging to the *Borrelia burgdorferi sensu lato* complex and relapsing fever *Borrelia* group have been reported from *Ixodes granulatus* collected from rodents [11, 12] and *Haemaphysalis hystricis* [13], respectively. Further, *Coxiella burnetii* was detected from *Dermacentor steini* and *H. hystricis* [14]. Studies on feeding and questing ticks collected from a forest reserve area, farms and indigenous settlements revealed the presence of bacterial species belonging to *Anaplasmatacea* and *Rickettsiaceae* that were closely related to the spotted fever group in *Amblyomma, Dermacentor* and *Haemaphysalis* spp. of ticks [8, 9, 15]. However, the potential of detected bacteria in causing zoonotic diseases in humans and animals is still largely unknown and requires further investigation.

In addition, ticks also harbour a high abundance of symbiotic and commensal microorganisms that can be obligate or facultative. *Coxiella *-like and *Francisella *-like endosymbionts (hereafter *Coxiella *-LE and *Francisella *-LE) are two well-established non-pathogenic microorganisms, which are essential for tick survival and development [16, 17]. Some known facultative endosymbionts such as *Arsenophonos*, *Rickettsia *, *Spiroplasma* and *Wolbachia* are commonly present in arthropods and have roles in the physiology of ticks [18]. These endosymbionts have also been described in ticks [19–23]. Moreover, ticks could acquire microorganisms via the environment and blood meals, and these factors could add to the complexity of the tick-associated bacterial ecologies [24, 25]. Previously, ontogeny stage and sex were among the variables shaping the bacterial community in ticks [26, 27]. Furthermore, the interaction between non-pathogenic and pathogenic microorganisms in ticks has gained substantial interest as it can be a fundamental tool for disease control measures. For instance, Abraham *et al*. [28] revealed the capability of *A. phagocytophilum* in modulating the gut microbiota of *Ixodes scapularis* ticks to favour its colonization. Narasimhan *et al*. [29] also demonstrated the link between gut microbiota and *Borrelia burgdorferi* colonization in *I. scapularis* ticks. It was also reported that shifting between pathogenic and non-pathogenic forms could occur for some bacterial genera as an evolutionary process due to ecological and epidemiological factors [30]. Hence, revealing the tick microbiome on top of pathogen detection has become a new trend.

Studies employing next-generation sequencing (NGS) to elucidate tick symbionts, pathogens and the interaction between the two are limited in Malaysia, with only two studies to date. The bacterial communities from three *Haemaphysalis* ticks (*H. hystricis*, *H. wellingtoni* and *H. bispinosa*) collected from domestic animals such as dogs, cats and chickens have been reported by Khoo *et al*. [31]. Another study presented the bacterial communities for *Haemaphysalis*, *Dermacentor* and *Amblyomma* spp. collected from wild boars in the peninsula [32]. Both studies targeted bacterial 16S rDNA hypervariable region V6 and focused on only one or two metrics for alpha and beta diversity analyses [31, 32]. Overall, the information for tick microbiomes in large parts of Malaysia is still unknown.

In this study, we selected primary forests and an oil palm plantation in Sarawak, Malaysian Borneo, as our study sites. Ticks were collected by flagging the vegetation and from rodent hosts. We targeted the microbiome in the collected tick species using NGS and bioinformatics. We further identified the potential symbionts and characterized species of potential pathogens detected for each tick species. We also explored the factors that may contribute to the difference in microbial composition and richness in ticks. Together, this was our first attempt to outline the tick microbiome and tick-borne pathogens (TBPs) from Sarawak state, which will provide the direction of upcoming research on TBP control strategies.

## Methods

### Tick sampling and identification

Ticks were collected in Sarawak, Malaysian Borneo, from protected primary forests, Gunung Gading National Park (1.69° N 109.85 °E) and Kubah National Park (1.61° N 110.20° E) in November 2018; and from an oil palm plantation (3.36° N 113.69° E) in March 2019. Questing ticks were collected by dragging white flannel cloths over the forest floor, and feeding ticks were removed from rodents that were trapped during the sampling period. All ticks were kept separately in 70 % ethanol and stored at −20 °C until sample processing and DNA extraction.

Ticks were morphologically identified to their genera or species levels based on taxonomic keys [33–37]. Molecular identification was performed using the previously published primers [38] (Table S1, available in the online version of this article) for the amplification of an approximately 400 bp fragment of the mitochondrial 16S rDNA partial sequence using one leg from the ticks by using the hot alkaline extraction method previously described by Mtambo *et al*. [39] with some modifications. Briefly, the tick leg was incubated at 95 °C for 10 min after adding 10 µl of 100 nM of sodium hydroxide, followed by addition of 2 µl Tris-hydrochloride buffer (pH 7.0). A total of 209 feeding and questing ticks of different developmental stages and statuses from *I. granulatus* (*n*=32), *H. hystricis* (*n*=36)*, Haemaphysalis shimoga* (*n*=109), *Dermacentor compactus* (*n*=4), *D. steini* (*n*=24) and *Dermacentor atrosignatus* (*n*=4) were used for this study. Details of the tick samples and collection sites are provided in Table 1.

**Table 1.** Tick samples collected from Gunung Gading National Park (GGNP), Kubah National Park (KNP) and an oil palm (OP) plantation

	Sampling period					
	November 2018		March 2019		Feeding status	
Tick species	GGNP	KNP	OP	Total	Feeding	Questing
***Ixodes granulatus***						
Adult male (AM)	n/a	n/a	n/a	n/a	n/a	n/a
Adult female (AF)	3	9	10	22	22	n/a
Nymph (N)	n/a	2	3	5	5	n/a
Larva (L)	n/a	n/a	5	5	5	n/a
Total	3	11	18	32	32	n/a
***Haemaphysalis hystricis***						
Adult male (AM)	n/a	n/a	n/a	n/a	n/a	n/a
Adult female (AF)	n/a	n/a	n/a	n/a	n/a	n/a
Nymph (N)	n/a	n/a	n/a	n/a	n/a	n/a
Larva (L)	1	n/a	35	36	4	32
Total	1	n/a	35	36	4	32
***Haemaphysalis shimoga***						
Adult male (AM)	n/a	n/a	21	21	n/a	21
Adult female (AF)	n/a	n/a	27	27	n/a	27
Nymph (N)	n/a	n/a	21	21	18	3
Larva (L)	n/a	n/a	40	40	23	17
Total	n/a	n/a	109	109	41	68
***Dermacentor compactus***						
Adult male (AM)	1	1	n/a	2	n/a	2
Adult female (AF)	1	1	n/a	2	n/a	2
Nymph (N)	n/a	n/a	n/a	n/a	n/a	n/a
Larva (L)	n/a	n/a	n/a	n/a	n/a	n/a
Total	2	2	n/a	4	n/a	4
***Dermacentor steini***						
Adult male (AM)	n/a	n/a	n/a	n/a	n/a	n/a
Adult female (AF)	5	2	n/a	7	3	4
Nymph (N)	n/a	n/a	n/a	n/a	n/a	n/a
Larva (L)	1	n/a	16	17	1	16
Total	6	2	16	24	4	20
***Dermacentor atrosignatus***						
Adult male (AM)	2	n/a	n/a	2	n/a	2
Adult female (AF)	1	1	n/a	2	n/a	2
Nymph (N)	n/a	n/a	n/a	n/a	n/a	n/a
Larva (L)	n/a	n/a	n/a	n/a	n/a	n/a
Total	3	1	n/a	4	n/a	4
**Total**				**209**	81	128

### Sample preparation and bacterial 16S rRNA gene amplification

After identification, ticks were washed with sterile PBS and crushed with a Micro Smash MS-100R (TOMY) for 30 s at 2500 r.p.m. DNA extraction was performed using a Wizard Genomic DNA Purification Kit (Promega), as specified in the manufacturer’s protocol for animal tissue. All ticks, including the nymph and larval stages, were processed as individual samples. Mock extraction with reagents and beads was also prepared in parallel as the control. Extracted DNA was stored at −20 °C until library preparation and sequencing.

We targeted the 16S rRNA gene V3–V4 hypervariable regions, and sample preparation was performed following the procedure in the Illumina 16S Metagenomic Sequencing library preparation manual (Illumina). The targeted region was amplified by PCR with the primer set (338F and 806R) as in Klindworth *et al*. [40]. A total volume of 25 µl PCR mixture was prepared with 12.5 µl of 2 ×KAPA HiFi HotStart ReadyMix (KAPA Biosystems), 1 µM of each forward and reverse primer, and 2 µl of the extracted DNA. Negative control PCRs were prepared using molecular-grade water in place of DNA samples, and mock DNA extractions were also subjected to PCR. Amplification was run using the following thermal cycling conditions: an initial denaturation at 95 °C for 3 min, followed by 35 cycles of 95 °C for 30 s, 55 °C for 30 s and 72 °C for 30 s, followed by a final extension for 5 min at 72 °C. The amplicon PCR products were electrophoresed on a 1.2 % agarose gel with Midori Green Direct DNA stain (Nippon Genetics) and visualized with a BLooK LED transilluminator (GeneDireX) for the expected 460 bp fragment size.

### Library preparation and sequencing

Amplified PCR products were purified via Agencourt AMPure XP beads (Beckman Coulter Life Sciences). A Nextera XT Index Kit (Illumina) was used to provide unique dual indices for each purified sample. The purification step was repeated, and the size integrity of the amplicons was verified on a Bioanalyzer. Finally, all samples were quantified and pooled in equimolar concentrations, and paired-end sequencing was conducted on an Illumina MiSeq platform using a MiSeq v3 reagent kit (600-cycle, 300 bp, paired-end; Illumina). The combined library included negative controls, which later were used as a standard to identify and remove suspected contaminants during the data analysis. Library preparation, including sample purifications and high-throughput sequencing, was conducted at the National Institute for Environmental Studies (NIES, Ibaraki, Japan).

### Data analysis

Microbiome data sequences were analysed in Quantitative Insights in Microbial Ecology 2 (QIIME2 2019.10) [41]. Illumina Fastq sequence data were demultiplexed and quality-filtered using the q2‐demux plugin followed by denoising with DADA2 [42], then assigned to amplicon sequence variants (ASVs). Potential contaminants were then identified using the Decontam package (version 1.16.0) [43] in R (version 4.0.2, Core R Team, 2020) by the frequency method with a threshold of 0.4 and checked manually with reference to the negative controls before filtering out using the QIIME2 sequence identifiers. Next, taxonomy was assigned using Greengenes 13_8 99 % reference sequences [44]. A total of 6969 features including the unidentified sequences and those identified as chloroplast and in negative controls were removed as well as bacterial sequences not assigned to phylum level. Paired-end reads were aligned with MAFFT [45] and used to reconstruct a rooted phylogenetic tree with FastTree2 [46].

Diversity analysis was performed on the decontam-filtered feature table at the species level in QIIME2 after samples were rarefied for sufficient sequencing depth for the observed number of ASVs from all samples and we removed samples with low sequence read (i.e. <100) counts. Four alpha-diversity metrics: Shannon’s diversity, observed features, Faith’s Phylogenetic Diversity (Faith’s PD) [47] and Pielou’s evenness, and four beta-diversity metrics: weighted UniFrac [48], unweighted UniFrac [49], Jaccard distance and Bray–Curtis dissimilarity, were quantified using QIIME2. We calculated alpha diversity based on four metrics by exporting and visualizing the results in R (version 2.13.0) using the qiime2R (version 0.99.6), ggplot2 (version 3.3.6) and phyloseq (version 1.42.0) packages [50]. We estimated the statistical differences in alpha diversities among tick species using a linear mixed-effects (LME) model using the nlme package (version 3.1–157) in R. For all ticks, the response variable was alpha diversity with tick species and status as fixed effect variables, and location as a random variable. We also performed analysis using only adult ticks to eliminate the variation due to different developmental stages, in which tick species was set as a fixed variable and location as the random variable. To understand the effect of developmental stage and feeding status variations on the alpha diversity of the microbiome, we fitted alpha diversity as a response variable and stage or status within *Haemaphysalis shimoga* ticks as a fixed effect variable. Modelling was performed using the stats package (version 4.2.1) in R [51]. Next, the significance of beta diversity was tested by permutational multivariate analysis of variance (PERMANOVA) [52] using 999 permutations. Principal coordinate analysis (PCoA) was plotted based on the four distance metrics using the R package phyloseq (version 1.42.0) [50] to visualize the differences.

In addition, we visualized the differential abundance of the taxonomic groups using the taxa_heatmap function in the qiime2R package (version 0.99.6) in R (version 2.13.0). Further, we conducted analysis of composition of microbiomes (ANCOM) [53] to determine the dissimilarity among different tick species and the effect of developmental stage and feeding status for *H. shimoga*. We ran the linear discriminant analysis effect size (LEfSe) using the Huttenhower lab Galaxy pipeline [54] to test this dissimilarity in the context of relative abundances. Additionally, for some indicated tick species, pairwise analysis was performed to test the difference between the ticks that harboured a specific bacterial species and that were negative (e.g. *Borrelia *-positive vs. *Borrelia *-negative *I. granulatus*).

### PCR amplification and bacterial characterization

PCRs were performed to characterize notable bacterial species detected in tick samples through the NGS screening. The bacteria *Anaplasma, Ehrlichia, Bartonella, Coxiella, Francisella* and *Rickettsia* were targeted in the PCR amplification and sequencing. The details of all primers used for the bacteria species identification are described in Table S1.

PCRs targeting the citrate synthase gene (*gltA*), which amplified a 694 bp fragment, and cell division protein gene (*ftsZ*), which amplified a 900 bp fragment of *Bartonella,* were conducted in semi-nested and single PCR, respectively. For *Francisella* species characterization, a fraction of the T-cell epitope gene (*tul4*) and 16S rDNA of *Francisella* were targeted in a single PCR to amplify 248 bp and 1 kb fragments, respectively. Single PCRs were conducted for *Rickettsia* species characterization by targeting six genes: *gltA* gene for 580 bp, outer membrane A gene (*ompA*) for 542 bp, outer membrane protein B gene (*ompB*) for 816 bp, 17 kDa common antigen gene (*htrA*) for 550 bp, 16S rDNA for 1.3 kb and surface cell antigen-4 gene (*Sca4*) for 928 bp fragment. All PCRs for *Bartonella *, *Francisella* and *Rickettsia* were conducted using *Ex Taq* Hot Start Version (Takara Bio) in a reaction mixture of 20 µl. The conditions used in the PCR assays were as follows: 35 or 40 cycles of denaturation at 94 °C for 30 s, annealing temperature according to each respective primer set for 30 s, and extension at 72 °C for 30 s, 60 s or 90 s depending on the targeted amplicon size.

Next, to characterize the species of *Anaplasma, Ehrlichia* and *Coxiella *, we used Tks Gflex DNA Polymerase (Takara Bio) with a 25 µl reaction mixture preparation. Nested PCR was conducted for *Anaplasma* and *Ehrlichia* by amplifying a 1.3 kb fragment of 16S rDNA of *Anaplasmatacea* with the following conditions: initial denaturation at 95 °C for 3 min, followed by 40 cycles of denaturation step at 95 °C for 30 s, 48 or 54 °C of annealing for 30 s, and extension at 68 °C for 90 s, with a final extension at 68 °C for 5 min. A total of five genes were used for *Coxiella* species characterization, which included chaperone protein DnaK gene (*dnaK*) for 512 bp, chaperone protein GROEL gene (*groEL*) for 619 bp, β subunit of bacterial RNA polymerase gene (*rpoB*) for an estimate of 550 bp, 16S rDNA for an estimate of 1 kb and large ribosomal subunit (23S rDNA) for a 583–867 bp fragment. DNA of *Coxiella* was amplified with nested or semi-nested PCRs, with the following conditions: initial denaturation at 94 °C for 1 min, followed by 40 cycles of denaturation at 98 °C for 10 s, 54 or 56 °C of annealing for 15 s, and extension at 68 °C for 1 min, with a final extension at 68 °C for 5 min.

Finally, the amplicon size was verified with electrophoresis and visualized as described above. Sanger sequencing was performed on the successfully amplified samples using the BigDye Terminator version 3.1 Cycle Sequencing Kit (Applied Biosystems). The obtained sequencing products were analysed on an ABI Prism 3130X genetic analyzer (Applied Biosystems), as per the manufacturer’s instructions. The resulting sequences were assembled and trimmed using the ATGC software version 9.0.0 (GENETYX) and compared with the sequences available in the public databases using the Nucleotide Basic Local Alignment Search Tool (BLASTn) (https://blast.ncbi.nlm.nih.gov/Blast.cgi).

## Results

**Fig. 1.**
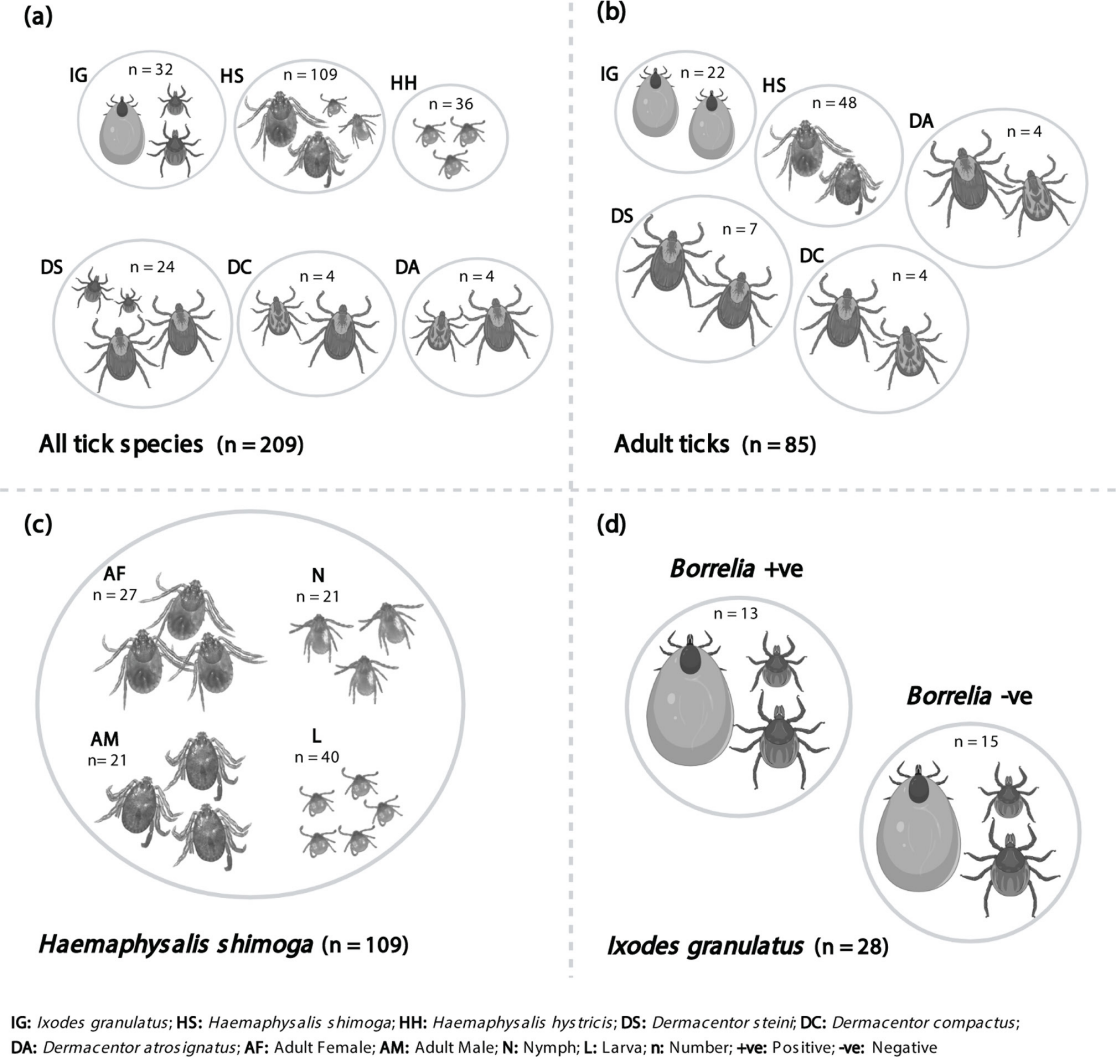
Data analyses were conducted as follows: (**a**)all tick species regardless of the developmental stage and status; (**b**)adult ticks after removing nymphs and larvae; (**c**)*Haemaphysalis shimoga,* which had a comprehensive sample size for all developmental stages, were analysed for different developmental stages and feeding statuses; and (**d**)*Ixodes granulatus,* which were positive for *Borrelia* spp., were compared with negative individuals. This figure was created using Biorender.com.

A total of 6 110 903 raw paired-end reads were obtained from the Illumina MiSeq sequencer. Sequences obtained were demultiplexed and quality filtered, resulting in 509 467 high-quality reads assigned to 9287 features retained after the DADA2 quality control analysis. Among 209 ticks from six species of three genera (Table 1), four samples were excluded from the analysis due to the significantly low number of obtained sequences. Tick samples were categorized into four groups for microbial analysis (Fig. 1). The first group included all ticks from their available developmental stages. The second group consisted of only the adult-stage ticks because not all tick species collected had all developmental stages represented (*H. hystricis* excluded). Next, since *H. shimoga* had the most comprehensive sample structure, we examined the effect of different developmental stages and feeding statuses (Fig. S1) on the microbiome for this species. Additionally, the effect of *Borrelia* infection on the tick microbiome was also examined for *I. granulatus* (Fig. 1).

### Tick species microbial variations regardless of developmental stage

**Fig. 2.**
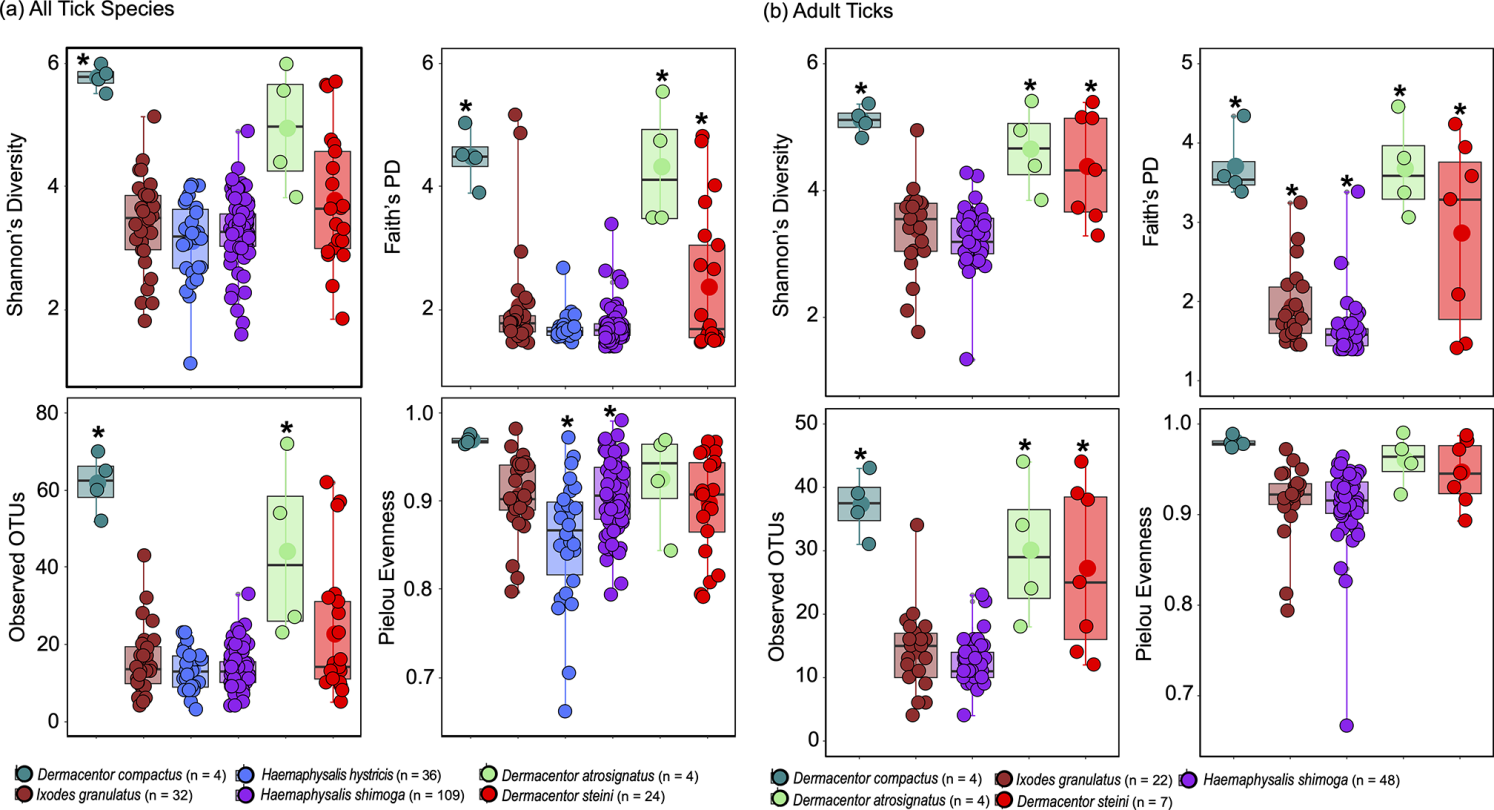
Alpha diversity analyses for (**a**)all tick species and (**b**)adult tick groups after decontam filtering. All analysis of significance was done by an LME in R software. Significance is shown with an asterisk (*) in the figures. (**a**)All tick species: Shannon’s diversity showed that microbiome diversity of *Dermacentor compactus* was significantly different from *Ixodes granulatus* and *Haemaphysalis hystricis* (*P*<0.01) and *Haemaphysalis shimoga* (*P*<0.05). In Faith’s PD, the genus *Dermacentor* showed a highly significant difference (*P*<0.01 or *P*<0.001) in microbiome diversity compared to other tick genera. In observed features, both *D. compactus* (*P*<0.01 or *P*<0.001) and *D. atrosignatus* (*P*<0.05) showed significant differences from other tick species. There was a highly significant difference (*P*<0.001) in Pielou's evenness between *H. hystricis* and *H. shimoga*. (**b**)Adult ticks: the genus *Dermacentor* showed a highly significant difference in microbiome diversity compared to *I. granulatus* and *H. shimoga* in Faith’s PD (*P*<0.001) and observed features (*P*<0.01 or *P*<0.001), and significant (*P*<0.05) and highly significant (*P*<0.01 or *P*<0.001) differences in Shannon’s diversity. Furthermore, microbiome diversity between *I. granulatus* and *H. shimoga* was highly significant (*P*<0.01) in Faith’s PD. In the adult tick group, *H. hystricis* were excluded from the analyses.

Microbial diversity analysis revealed significant differences among tick species in four metrics regardless of the tick developmental stages (Table S2 and Fig. 2). The genus *Dermacentor* showed the highest microbial diversity in Faith’s PD (*P*<0.001), in observed features (*P*<0.05 and *P*<0.001) and Shannon’s diversity (*P*<0.05) (Fig. 2a, b). Next, *I. granulatus* had significantly higher microbial diversity than *H. shimoga* as calculated by Faith’s PD (*P*<0.05) (Fig. 2b). When comparing the microbial diversity between the three species of *Dermacentor*, the microbiome of *D. steini* was significantly less diverse than that of *D. compactus* based on the estimated observed features (*P*<0.05) and in Faith’s PD (*P*<0.001), and of *D. atrosignatus* by Faith’s PD (*P*<0.001) (Fig. 2a). However, it was non-significant when only adult-stage *Dermacentor* was included in the analysis (Fig. 2b). ANCOM and pairwise LEfSe analyses also supported these findings and indicated that the genus *Dermacentor* displayed a greater number of taxonomic groups that were significantly more abundant than in other tick species, consistent with the alpha diversity results (Figs S2 and S3). Finally, species evenness was relatively high for all species examined, and a significant difference was observed between *H. hystricis* and *H. shimoga* (*P*<0.001) (Fig. 2a).

**Fig. 3.**
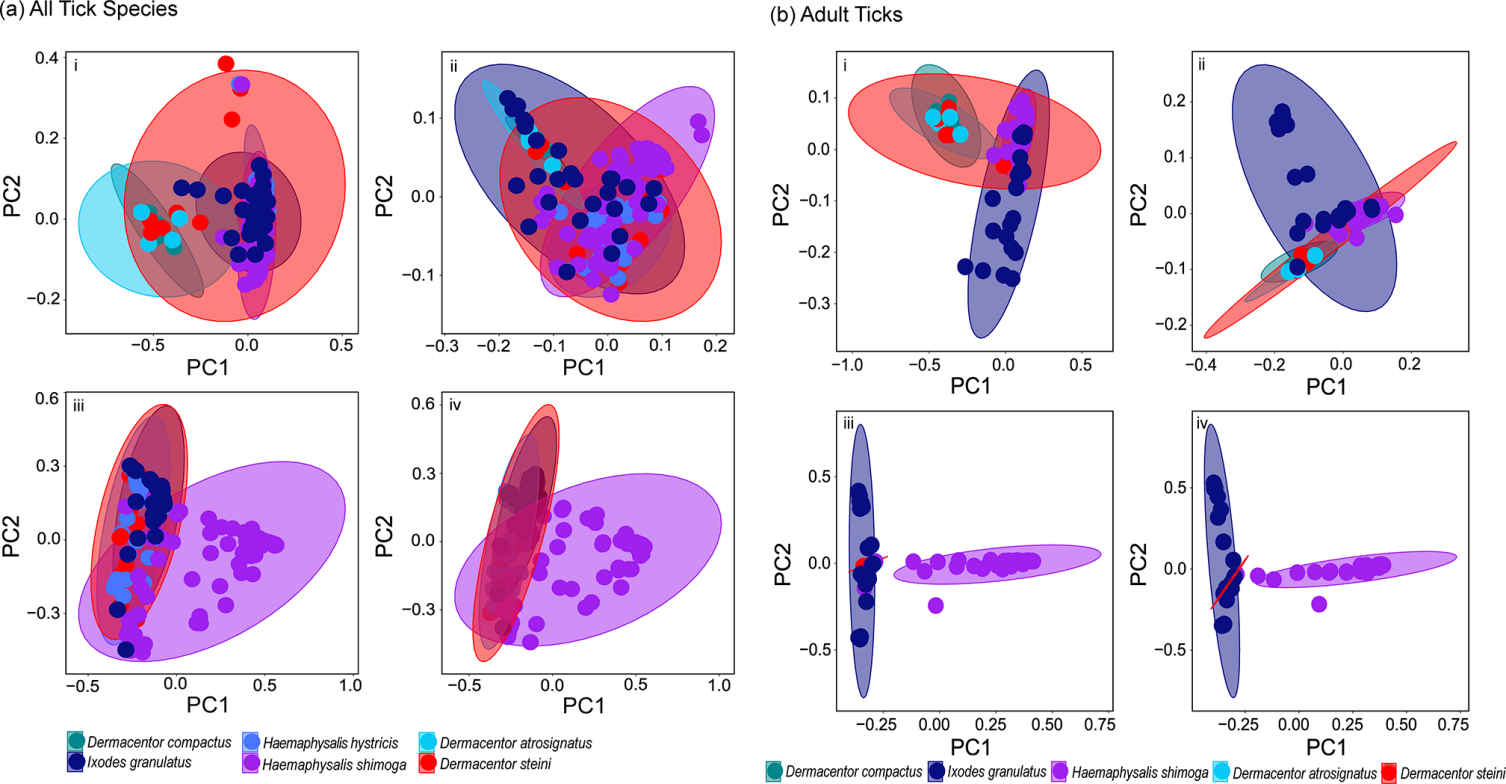
Beta diversity analyses for (**a**)all tick species and (**b**)adult tick groups. PCoA plots for (i) unweighted UniFrac distance, (ii) weighted UniFrac distance, (iii) Jaccard distance and (iv) Bray–Curtis dissimilarity.

Pairwise PERMANOVA comparisons for the beta diversity analyses revealed that microbial composition was significantly different between the tick species (Fig. 3a), except between *D. compactus*, *D. steini* and *D. atrosignatus* (Table S3). Consistently, results with only adult ticks showed significant differences between tick species, except for the three *Dermacentor* spp. Of all tick species, *H. shimoga* was significantly different from other tick species for all beta diversity metrics with the greatest pseudo-*F* values (*P*=0.001, d.f.=5) (Table S3). Adult *H. shimoga* ticks also formed a distinct cluster from *I. granulatus* and *Dermacentor* species in the Bray–Curtis dissimilarity and Jaccard distance plots (Fig. 3b). Meanwhile, *H. hystricis* also showed greater differences in unweighted and weighted UniFrac distances compared to *D. compactus* (pseudo-*F*=27.76 and 18.95; *P*=0.001, d.f.=5) and *D. atrosignatus* (pseudo-*F*=26.79 and 18.2; *P*=0.001, d.f.=5). No significant difference was observed among the three *Dermacentor* species (*P*>0.05, d.f.=5), and they clustered together as shown in unweighted and weighted UniFrac distance plots of adult ticks (Fig. 3b).

### Microbial composition and potential symbionts for each tick species

**Fig. 4.**
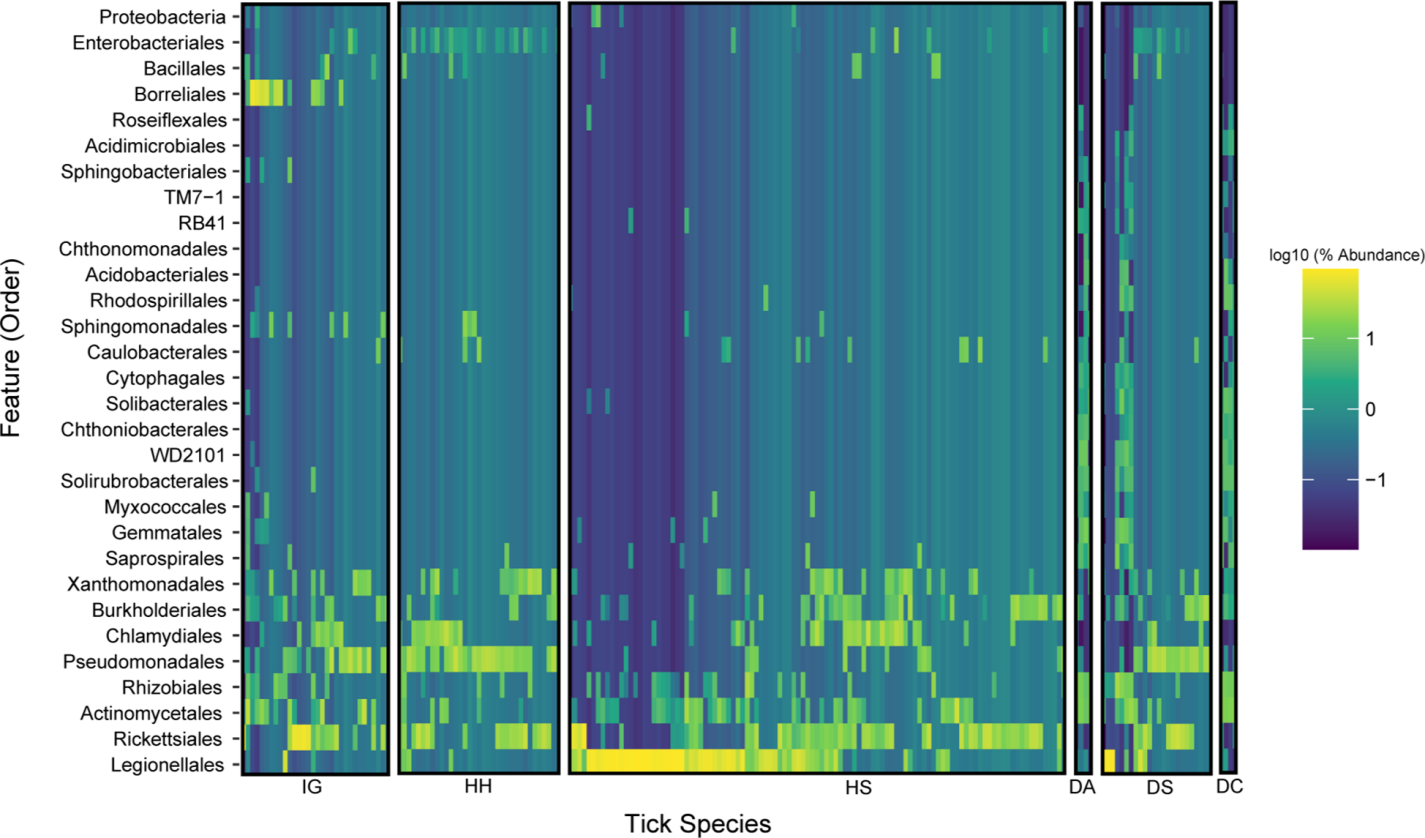
Heat map for all the tick species based on bacterial order level. IG: *Ixodes granulatus*; HH: *Haemaphysalis hystricis*, HS: *H. shimoga*, DA: *Dermacentor atrosignatus*, DS: *D. steini*, and DC: *D. compactus*.

**Fig. 5.**
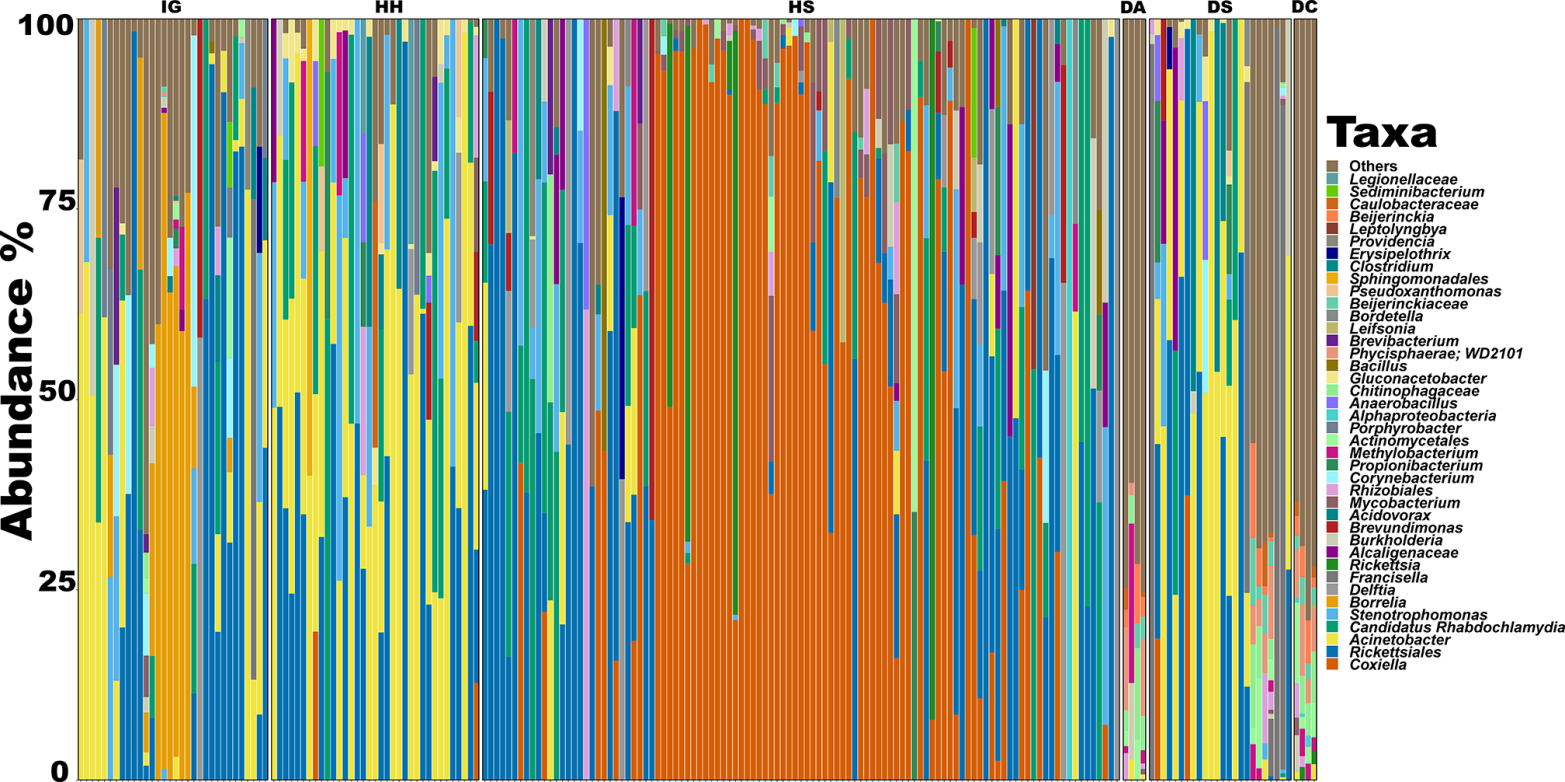
Relative abundance (%) of bacterial taxa identified in the microbiome of six species of ixodid ticks. The figure displays the most abundant 40 taxa individually with the remaining grouped together. Each bar represents the genus level of the bacterial taxa detected in one sample. IG: *Ixodes granulatus*; HH: *Haemaphysalis hystricis*, HS*: H. shimoga*, DA: *Dermacentor atrosignatus*, DS: *D. steini*, and DC: *D. compactus*.

Overall, the most abundant phylum identified in ticks was *Pseudomonadota* (76.77 %). Each of the remaining phyla accounted for less than 10%, and they were *Actinomycetota* (6.69 %), *Chlamydiota* (6.67 %), *Spirochaetota* (3.26 %), *Bacillota* (1.44 %), *Bacteroidota* (1.43 %) and *Planctomycetota* (1.12 %) (Table S4A). Notably, *Spirochaetota* were only present in *I. granulatus* and accounted for 18.9 % of its total relative abundance, which was characterized as *Borrelia yangtzensis *, a member of the *Borrelia burgdorferi sensu lato* complex, in our previous study for *Borrelia* screening [12]. A heat map (Figs 4, 5and S2) showed the taxonomy groups that were more abundantly represented in each tick species. For instance, *Rickettsiales* could be found in most tick species such as *I. granulatus*, *H. hystricis*, *H. shimoga* and *D. steini*, while order *Legionellales* was mainly distributed in *H. shimoga*. Differentially abundant taxonomy groups for each tick species were identified by pairwise LEfSe analyses. Taxonomic groups such as *Planctomycetota *, *Actinomycetota *, *Bacteroidota* and *Rhizobiales* were highly abundant in *D. compactus* and *D. atrosignatus*. In contrast, taxonomic groups such as *Gammaproteobacteria *, *Pseudomonadales *, *Legionellales* and *Francisellaceae* were more abundant in *D. steini* (Fig. S3). LEfSe results also indicated that *H. shimoga* harboured more bacterial taxa than *H. hystricis*, including *Coxiellaceae *, *Actinomycetales* and *Mycobacteriaceae* (Fig. S3). Additionally, while *Coxiellaceae* was the most abundant taxon found in *H. shimoga*, *Borreliaceae* was evident for *I. granulatus* and *Francisellaceae* for *D. steini*. *Pseudomonadales* and *Enterobacterales* were among the bacterial taxa found t greater abundance in *H. hystricis* when compared with other tick species (Fig. S3).

**Fig. 6.**
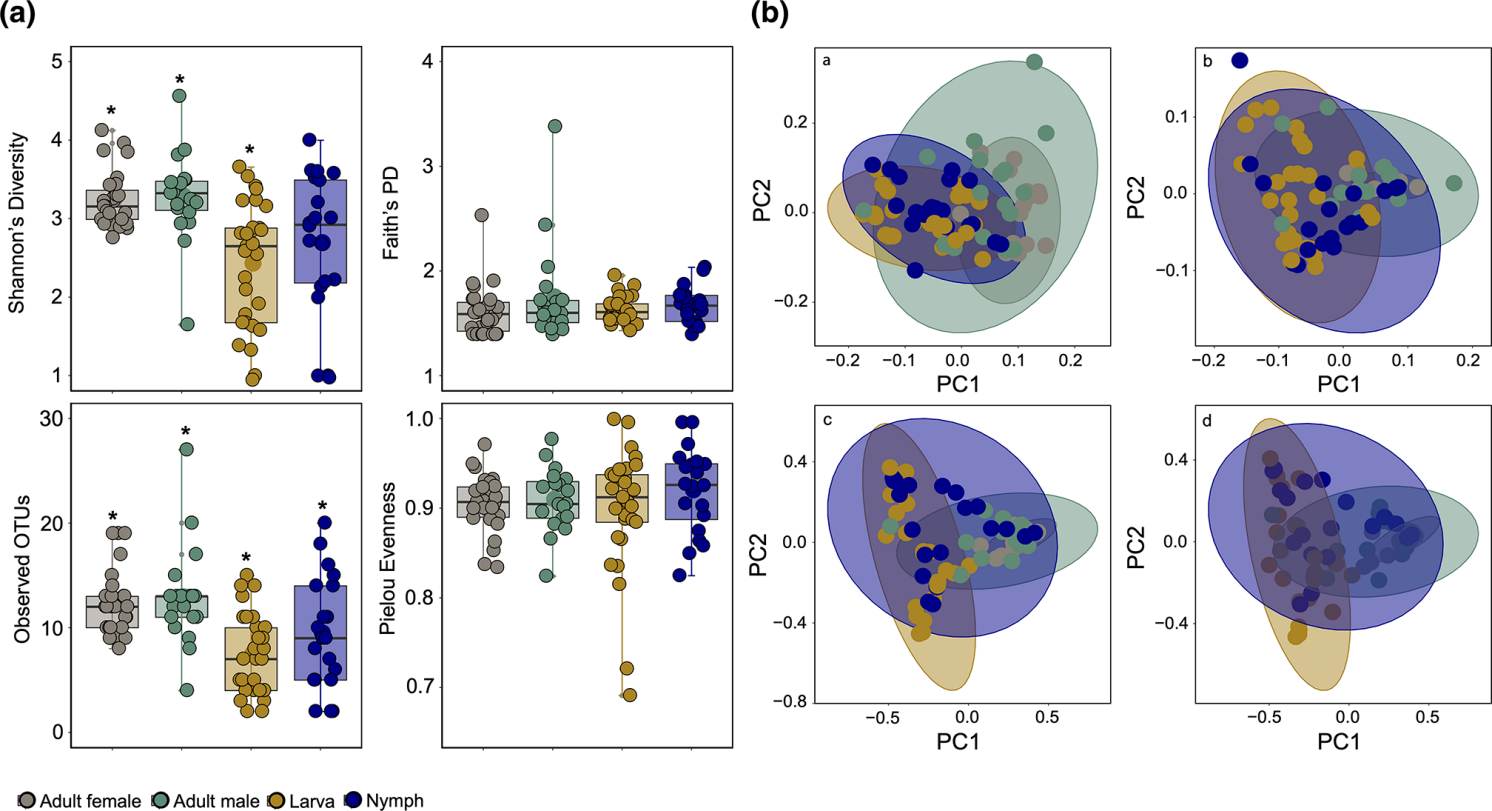
Alpha and beta diversity analyses for different developmental stages of *Haemaphysalis shimoga*. (**a**)Alpha diversity analyses. Significance is shown with an asterisk (*) in the figure. In Shannon’s diversity, the microbiome diversity of adult ticks was significantly different from larvae (*P*<0.01). In observed features, adult ticks were significantly different from larvae (*P*<0.001) and nymphs (*P*<0.05). (**b**)PCoA plots for (a)unweighted UniFrac distance, (b)weighted UniFrac distance, (c)Jaccard distance and (d)Bray–Curtis dissimilarity.

Potential symbionts were identified for all examined tick species except for *D. compactus* and *D. atrosignatus*. For *I. granulatus*, *Rickettsiales* (25.63 %) was the dominant bacterial taxon, and for *H. shimoga*, *Coxiella* accounted for 50 % of its total relative abundance (Table 2). Some tick species had more than one potential bacterial symbiont dominating; for instance, *Acinetobacter* and *Rickettsiales* were the dominant bacteria in *H. hystricis*. In *D. steini*, *Acinetobacter* (21.96 %), *Rickettsiale* s (17.90 %) and *Francisella* (17.87 %) were most abundant (Table 2). Meanwhile, in *D. compactus* and *D. atrosignatus*, most bacterial taxa were present with a relative abundance of less than 10 %. Furthermore, *Francisella* was not detected in *D. compactus* or *D. atrosignatus*, but we detected *Francisella* (2.25 %) in *I. granulatus* (Table S4B, C).

**Table 2.** Bacterial taxa in high relative abundance identified in *Ixodes granulatus, Haemaphysalis hystricis, Haemaphysalis shimoga* and *Dermacentor steini*

Tick species	Bacterial taxa	Relative abundance (%)
*Ixodes granulatus*	* Rickettsiales *	25.63
	* Borrelia *	18.9
	* Acinetobacter *	11.75
	*Candidatus* Rhabdochlamydia	6.36
*Haemaphysalis hystricis*	* Acinetobacter *	30.06
	* Rickettsiales *	25.3
	* Stenotrophomonas *	10.41
	*Candidatus* Rhabdochlamydia	10.02
*Haemaphysalis shimoga*	* Coxiella *	50
	* Rickettsiales *	13.69
	*Candidatus* Rhabdochlamydia	7.32
	* Stenotrophomonas *	5.2
*Dermacentor steini*	* Acinetobacter *	21.96
	* Rickettsiales *	17.9
	* Francisella *	17.87
	* Burkholderiales *	7.96

### Ontogenetic microbial variations of *Haemaphysalis shimoga*

Alpha diversity analysis of different developmental stages of *H. shimoga* ticks revealed that adult ticks showed significantly higher microbial diversity than nymphs and larvae by observed features (*P*<0.05 and *P*<0.001, respectively) and Shannon’s diversity (*P*<0.05) (Fig. 4a). However, microbial diversity was not significantly different between adult male and female ticks and between the nymph and larva of *H. shimoga* ticks. Pielou’s evenness analysis showed that the microbiota was evenly distributed in all *H. shimoga* developmental stages (Fig. 4a). Furthermore, the microbial composition of the adult females was significantly different (*P*=0.001) from that of nymphs and larvae, in which beta diversity analysis results showed distinct clusters between adult females and larvae in weighted UniFrac distance (pseudo-*F*=34.29, d.f.=3), Bray–Curtis dissimilarity (pseudo-*F*=24.62, d.f.=3) and Jaccard distance (pseudo-*F*=20.87, d.f.=3) plots of *H. shimoga* (Table S3; Fig. 4b). Similarly, adult male and larva *H. shimoga* ticks were significantly different (*P*=0.001) in microbial composition in unweighted (pseudo-*F*=5.84, d.f.=3) and weighted (pseudo-*F*=10.50, d.f.=3) UniFrac distance, Bray–Curtis dissimilarity (pseudo-*F*=8.57, d.f.=3) and Jaccard distance (pseudo-*F*=8.37, d.f.=3) analyses (Table S3). Adult male *H. shimoga* ticks were also significantly different from *H. shimoga* nymphs in unweighted (pseudo-*F*=3.52; *P*=0.001, d.f.=3) and weighted (pseudo-*F*=4.79; *P*=0.004, d.f.=3) UniFrac distance, Bray–Curtis dissimilarity (pseudo-*F*=3.53; *P*=0.002, d.f.=3) and Jaccard distance (pseudo-*F*=4.32; *P*=0.003, d.f.=3) (Table S3). There was a significant difference detected between the microbial composition of adult male and female *H. shimoga* ticks in unweighted (pseudo-*F*=1.91; *P*=0.03, d.f.=3) and weighted (pseudo-*F*=3.57; *P*=0.006, d.f.=3) UniFrac distance, Bray–Curtis dissimilarity (pseudo-*F*=2.65; *P*=0.004, d.f.=3) and Jaccard distance (pseudo-*F*=2.63; *P*=0.003, d.f.=3) analyses (Table S3). Additionally, nymphs and larvas of *H. shimoga* ticks were not greatly different in microbial composition, with unweighted (pseudo-*F*=1.65; *P*=0.126, d.f.=3) and weighted (pseudo-*F*=1.85; *P*=0.11, d.f.=3) UniFrac distance showed nosignificant difference, while Bray–Curtis dissimilarity (pseudo-*F*=2.13; *P*=0.006, d.f.=3) and Jaccard distance (pseudo-*F*=2.10; *P*=0.001, d.f.=3) revealed significance (Table S3).

**Fig. 7.**
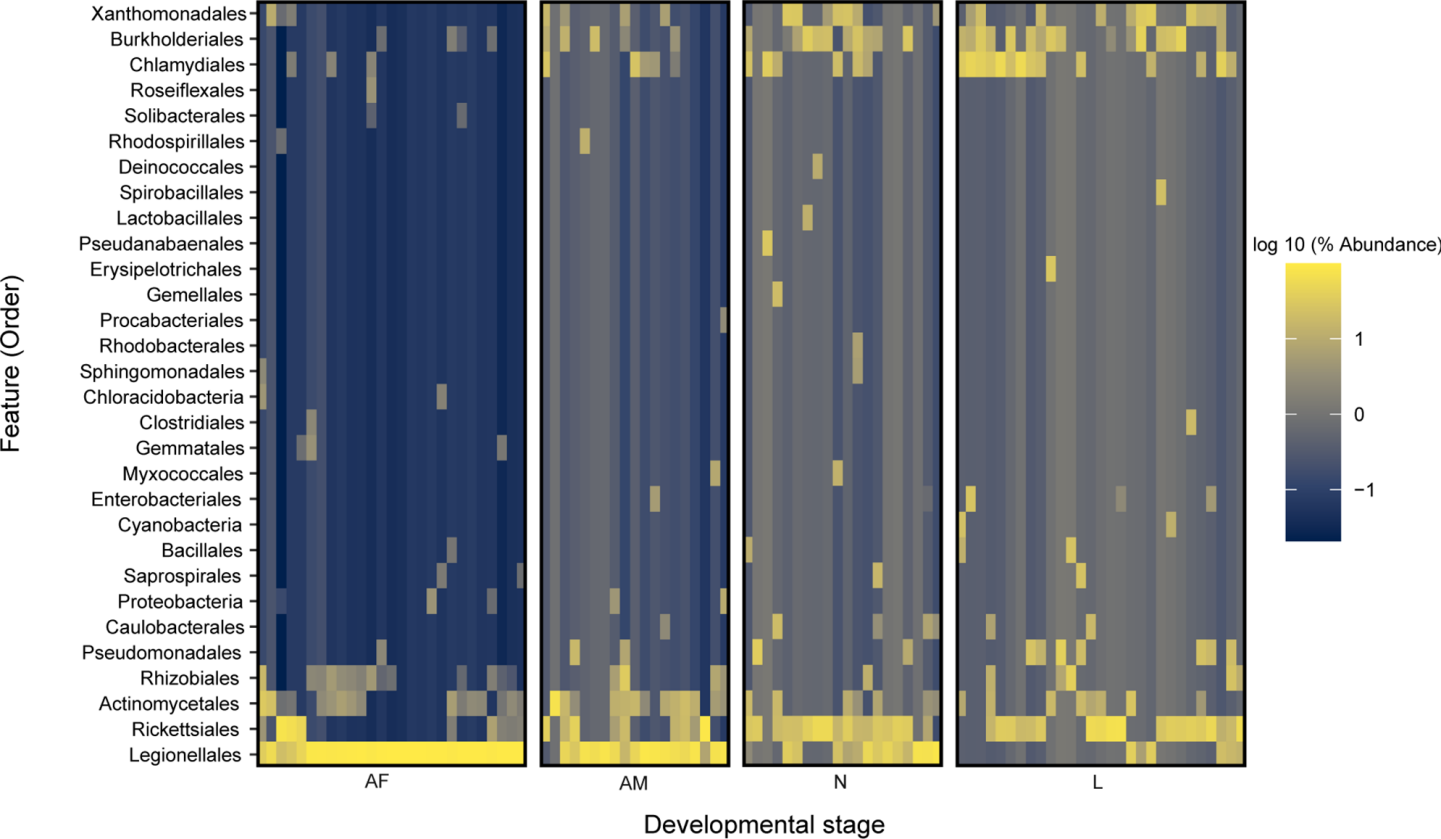
Heat map for different developmental stages of *Haemaphysalis shimoga* based on bacterial order level. AF: adult female, AM: adult male, N: nymph, and L: larva.

**Fig. 8.**
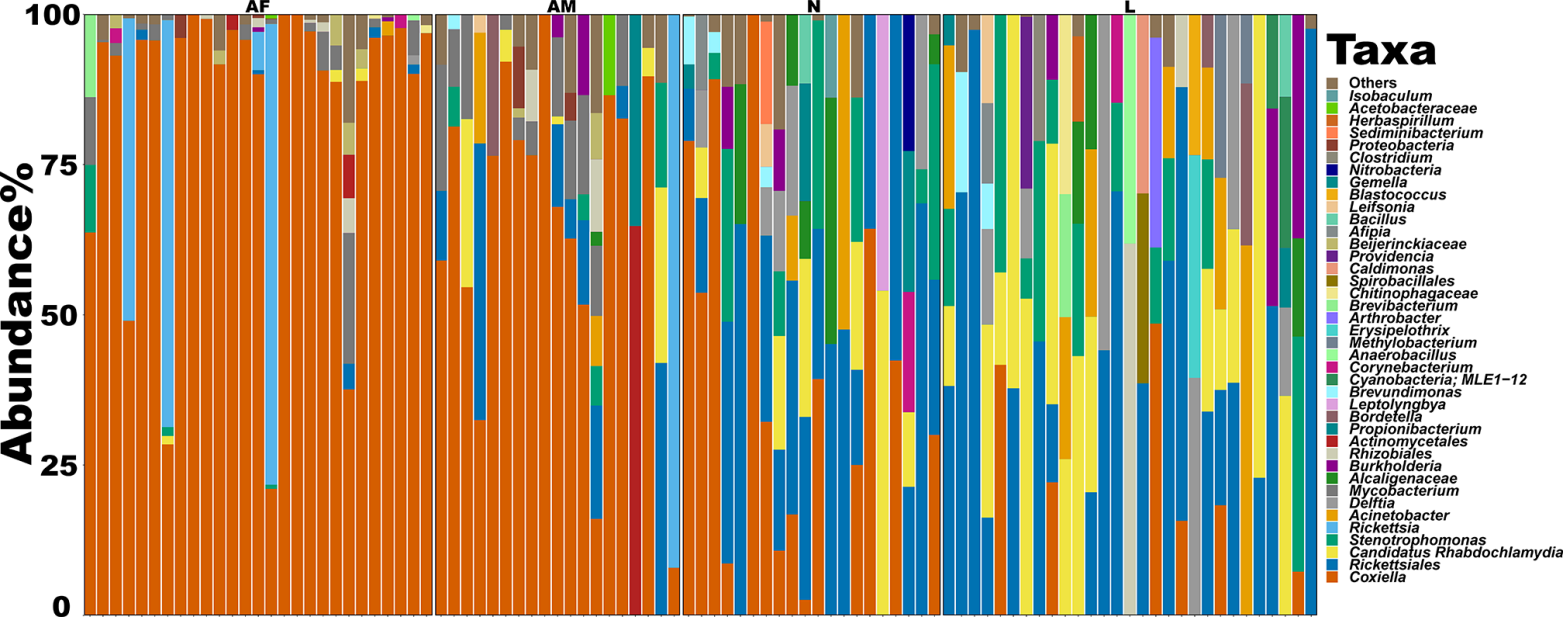
Relative abundance (%) of bacterial taxa identified in the microbiome of different developmental stages of *Haemaphysalis shimoga*. The figure displays the most abundant 40 taxa individually with the remaining grouped together. Each bar represents the genus level of the bacterial taxa detected in one sample. AF: adult female, AM: adult male, N: nymph, and L: larva.

**Fig. 9.**
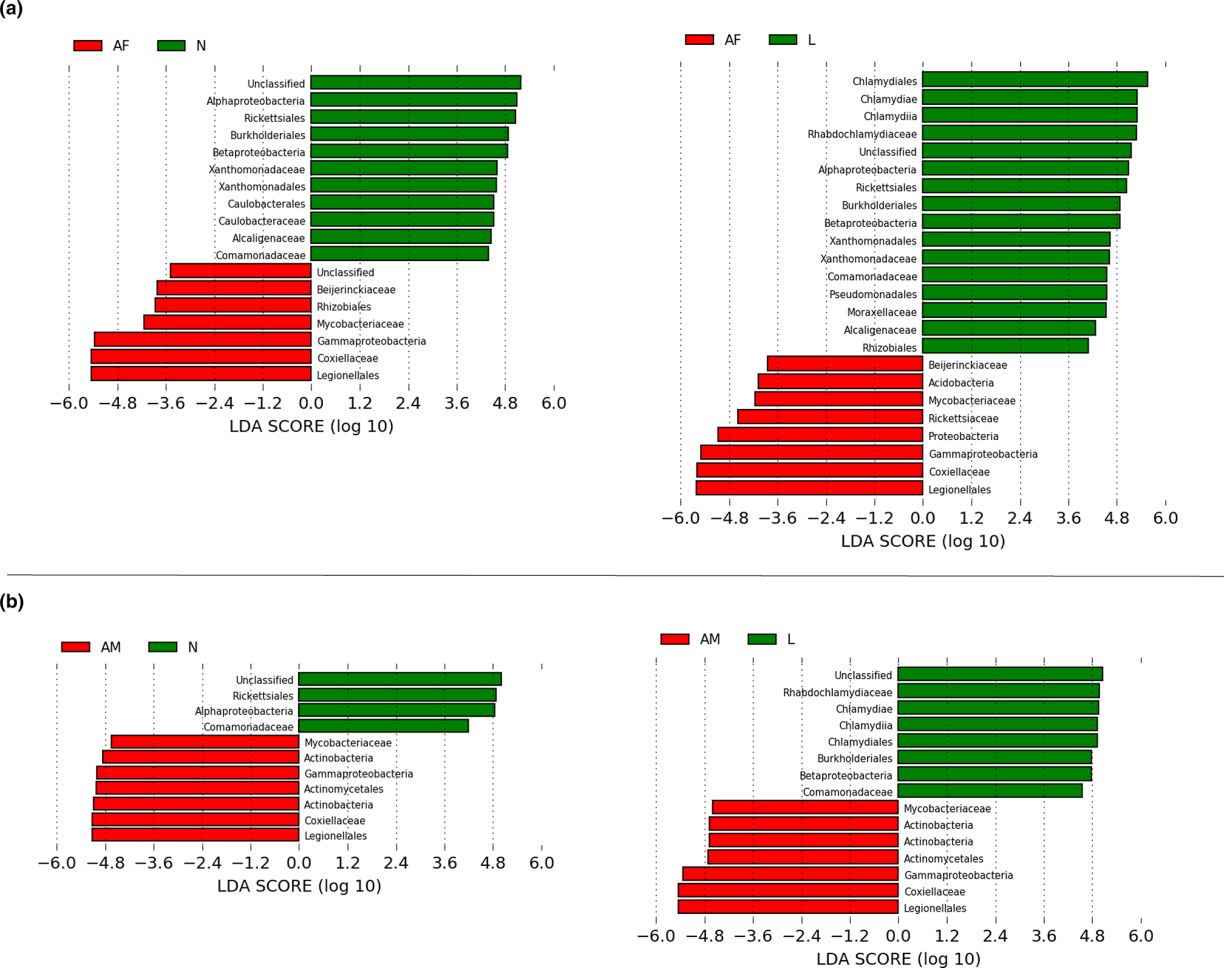
Pairwise LEfSe bar plots showing the bacterial taxa significantly different between different developmental stages of *Haemaphysalis shimoga*. (a) Differential abundance of bacteria between adult female (AF) and nymph (N)or larva (L).(b) Differential abundance of bacteria between adult male (AM) and nymph or larva.

Furthermore, ANCOM (Table S5) and pairwise LEfSe analyses revealed differential abundance for adult ticks with regard to *Coxiellaceae *, *Actinomycetota *, *Actinomycetales* and *Mycobacteriaceae *. In addition, *Rickettsiales *, *Burkholderiale* s, *Xanthomonadales* and *Caulobacterales* were significantly more abundant in nymphs than adult female ticks (Figs 7–9a), whereas *Rickettsiales* and *Comamonadaceae* were significantly more abundant in nymphs than adult male ticks (Fig. 9b). One phylum, three classes, six orders and five families were significantly more abundant in larvae when compared with adult female ticks, including orders *Chlamydiales* and *Burkholderiales *, which were also significantly more abundant than in adult male ticks (Fig. 9). This microbial difference observed was supported by the presence of potential symbionts in different developmental stages and sex. The genus *Coxiella* (order *Legionellales *) was found to dominate the adult ticks, and two potential symbionts were observed for nymphs and larvae (Table 3, Figs 7 and 8) . For instance, the presence of *Rickettsiales* and *Coxiella* in nymphs and *Rickettsiales* and *Candidatus* Rhabdochlamydia (order *Chlamydiales *) in larvae was noted. Contrary to nymphs and larvae, the order *Rickettsiales* was found in low relative abundance in adult ticks (Figs 7 and 8).

**Table 3.** Bacterial taxa in high relative abundance present in each developmental stage of *Haemaphysalis shimoga*

Developmental stage	Bacterial taxa	Relative abundance (%)
Adult male (AM)	* Coxiella *	62.04
	* Rickettsiales *	8.8
	* Mycobacterium *	7.01
	* Rickettsia *	5.12
Adult female (AF)	* Coxiella *	80.25
	* Rickettsia *	7.18
	* Rickettsiales *	2.42
	* Mycobacterium *	1.83
Nymph (N)	* Rickettsiales *	29.91
	* Coxiella *	25.8
	* Burkholderiales *	13.11
	* Stenotrophomonas *	9.6
Larva (L)	* Rickettsiales *	24.45
	*Candidatus* Rhabdochlamydia	21.92
	* Stenotrophomonas *	11.51
	* Coxiella *	8.09

### The effect of *Borrelia* infection on *Ixodes granulatus* microbiome

**Fig. 10.**
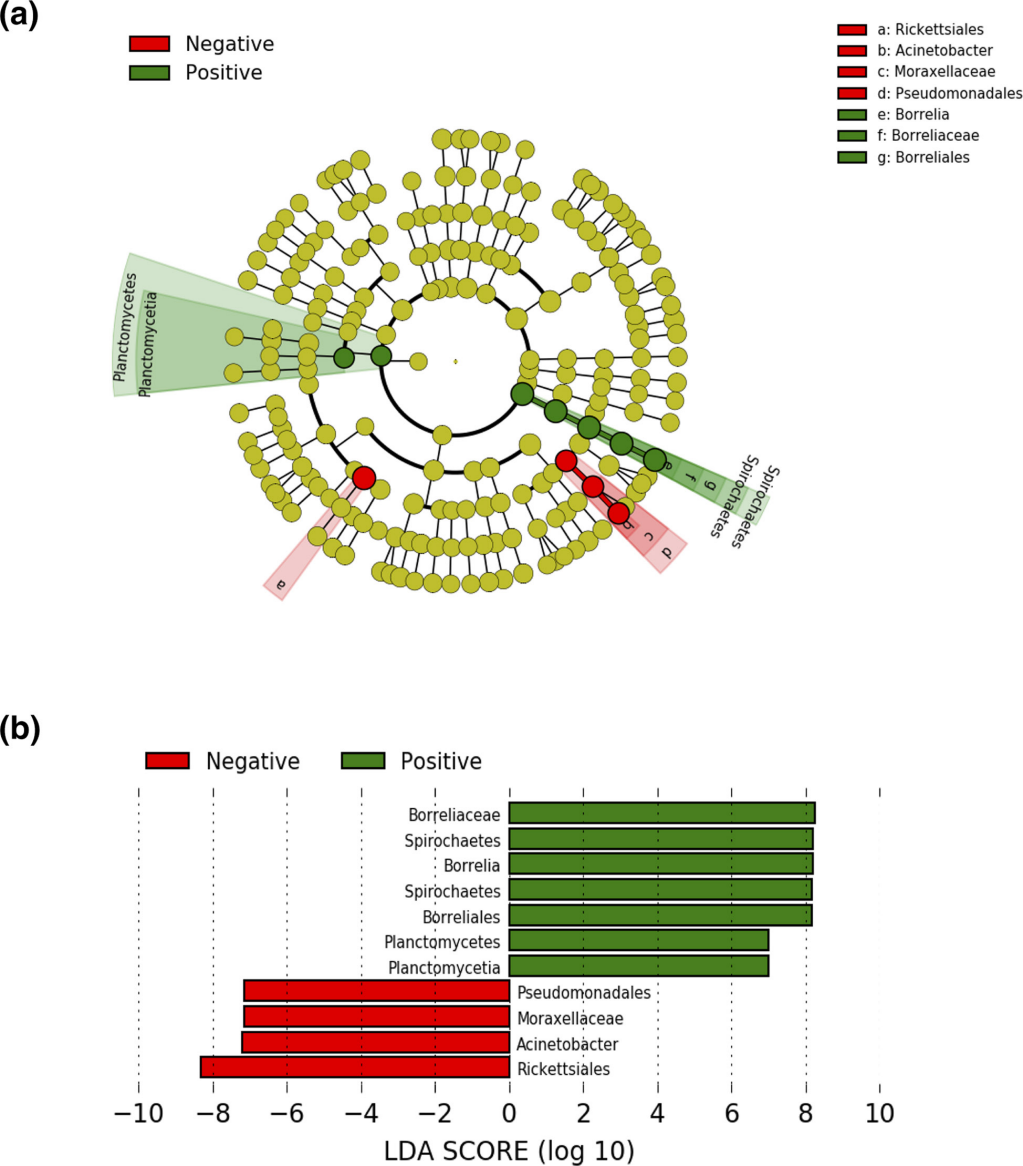
Pairwise LEfSe (a) cladogram and (b) bar plots showing bacterial taxonomy significantly different between *Borrelia *-positive and *Borrelia *-negative *Ixodes granulatus*.

Alpha and beta diversity analyses were conducted on 13 *Borrelia *-positive and 15 *Borrelia *-negative *I. granulatus* ticks. Based on PERMANOVA pairwise comparisons, the microbial composition between the positive and negative ticks was significantly different (*P*=0.001, *d.f.*=1) for unweighted and weighted UniFrac distance, Jaccard distance and Bray–Curtis dissimilarity analyses (Table S3). The difference was also depicted in PCoA plots with distinguishable clusters (Fig. S4). For the microbial diversity analyses, we detected significantly different values for Faith’s PD (*P*=0.006) (Fig. S4). Additionally, pairwise LEfSe analysis revealed the bacterial taxa with significant differential abundances for the positive and negative ticks. For instance, *Planctomycetot* a was found to be significantly more abundant in *Borrelia *-positive ticks, while *Borrelia *-negative ticks, on the other hand, had more abundant *Rickettsiales *, *Acinetobacter *, *Moraxellaceae* and *Pseudomonadales* (Fig. 10).

### Potential symbionts and pathogens characterized in ticks

The potential symbionts or pathogenic bacterial species detected by NGS were verified and characterized using conventional PCRs. The number of NGS- and PCR-positive ticks are shown in Table S6. Table 4 lists the sequence analysis results from the successfully amplified bacteria from their respective tick species and the targeted genes that were amplified by PCRs. Overall, bacterial species from six genera, *Coxiella *, *Francisella *, *Rickettsia *, *Anaplasma *, *Ehrlichia* and *Bartonella *, were identified from the ticks.

**Table 4.** Sequence analysis results for the bacterial genera *Coxiella, Francisella, Rickettsia, Anaplasma, Ehrlichia* and *Bartonella* in different tick species based on each successfully amplified gene

Bacteria	Genes	Tick species	BLASTn	Identity	Accession no.
* Coxiella *	16S rRNA	*Haemaphysalis hystricis*	Uncultured *Coxiella* sp. isolate S006 from *Haemaphysalis hystricis* (Malaysia)	99.8 % (1273/1275 bp)	LT009433
		*Haemaphysalis shimoga*	Bacteral symbiont of *Haemaphysalis shimoga* clone HSKY-3 (Thailand)	99.8 % (1275/1277 bp)	HQ287535
	23S rRNA	*Haemaphysalis hystricis*	* Coxiella burnetii* strain RSA439 from *Dermacentor andersoni* (USA)	92.0 %(496/539 bp)	CP040059
		*Haemaphysalis shimoga*	* Coxiella burnetii* strain RSA439 from *Dermacentor andersoni* (USA)	91.9 %(500/544 bp)	CP040059
	*GroEL*	*Haemaphysalis hystricis*	Uncultured *Coxiella* sp. clone PK179-183 from chicken tick (Thailand)	93.7 %(479/511 bp)	MG874468
		*Haemaphysalis shimoga*	Uncultured *Coxiella* sp. clone T3115 from *Ixodes uriae* (Canada)	86.7 %(494/570 bp)	KJ459059
	*rpoB*	*Haemaphysalis hystricis*	Uncultured *Coxiella* sp. isolate S002 from *Haemaphysalis hystricis* (Malaysia)	99.8 %(490/491 bp)	LT174612
		*Haemaphysalis shimoga*	Uncultured *Coxiella* sp. isolate S002 from *Haemaphysalis hystricis* (Malaysia)	89.9 %(438/487 bp)	LT174612
	*dnaK*	*Haemaphysalis shimoga*	* Coxiella* endosymbiont of *Ixodes* sp. isolate Isp1iso2 (Cote d'Ivoire)	86.9–87.1 % (391–392/450 bp)	KP985406
* Francisella *	*tul4*	*Ixodes granulatus*	* Francisella *-like endosymbiont strain FLE011 from *Hyalomma marginatum marginatum* (Bulgaria)	94.9 %(203/214 bp)	HQ705174
		*Dermacentor steini*	* Francisella *-like endosymbiont strain FLE011 from *Hyalomma marginatum marginatum* (Bulgaria)	94.9 %(203/214 bp)	HQ705174
	16S rRNA	*Dermacentor steini*	* Francisella* endosymbiont of *Dermacentor atrosignatus* isolate DASSD4 (Thailand)	99.9 %(1100/1101 bp)	KC170748
* Rickettsia *	*gltA*	*Haemaphysalis shimoga*	* Rickettsia heilongjiangensis* Sendai-58 from *Haemaphysalis concinna* (Japan)	100 %(537/537 bp)	AP019865
	*ompA*		* Rickettsia japonica* strain PMK (Thailand)	100 %(491/491 bp)	DQ909072
	*ompB*		* Rickettsia heilongjiangensis* Sendai-58 from *Haemaphysalis concinna* (Japan)	99.9 %(769/770 bp)	AP019865
	*htrA*		* Rickettsia heilongjiangensis* Sendai-58 from *Haemaphysalis concinna* (Japan)	100 %(495/495 bp)	AP019865
	*Sca4*		* Rickettsia heilongjiangensis* Sendai-58 from *Haemaphysalis concinna* (Japan)	100 %(888/888 bp)	AP019865
	16S rRNA		* Rickettsia heilongjiangensis* Sendai-58 from *Haemaphysalis concinna* (Japan)	100 %(1243/1243 bp)	AP019865
* Anaplasma *	16S rRNA	*Dermacentor atrosignatus*	* Anaplasma platys* isolate 2a×1 from sika deer (China)	98.9 %(1313/1327 bp)	KJ659044
* Ehrlichia *	16S rRNA	*Haemaphysalis shimoga*	* Ehrlichia* sp. EBm52 from *Rhipicephalus microplus* (Thailand)	98.9 %(1315/1330 bp)	AF497581
* Bartonella *	*gltA*	*Dermacentor steini*	* Bartonella rattimassiliensis* sp. nov. from European *Rattus norvegicus* (France)	97.8 %(703/719 bp)	AY515124
	*ftsZ*	*Ixodes granulatus*	* Bartonella tribocorum* strain MVT04 from human blood (France)	97.1 %(868/894 bp)	HG969192

**Fig. 11.**
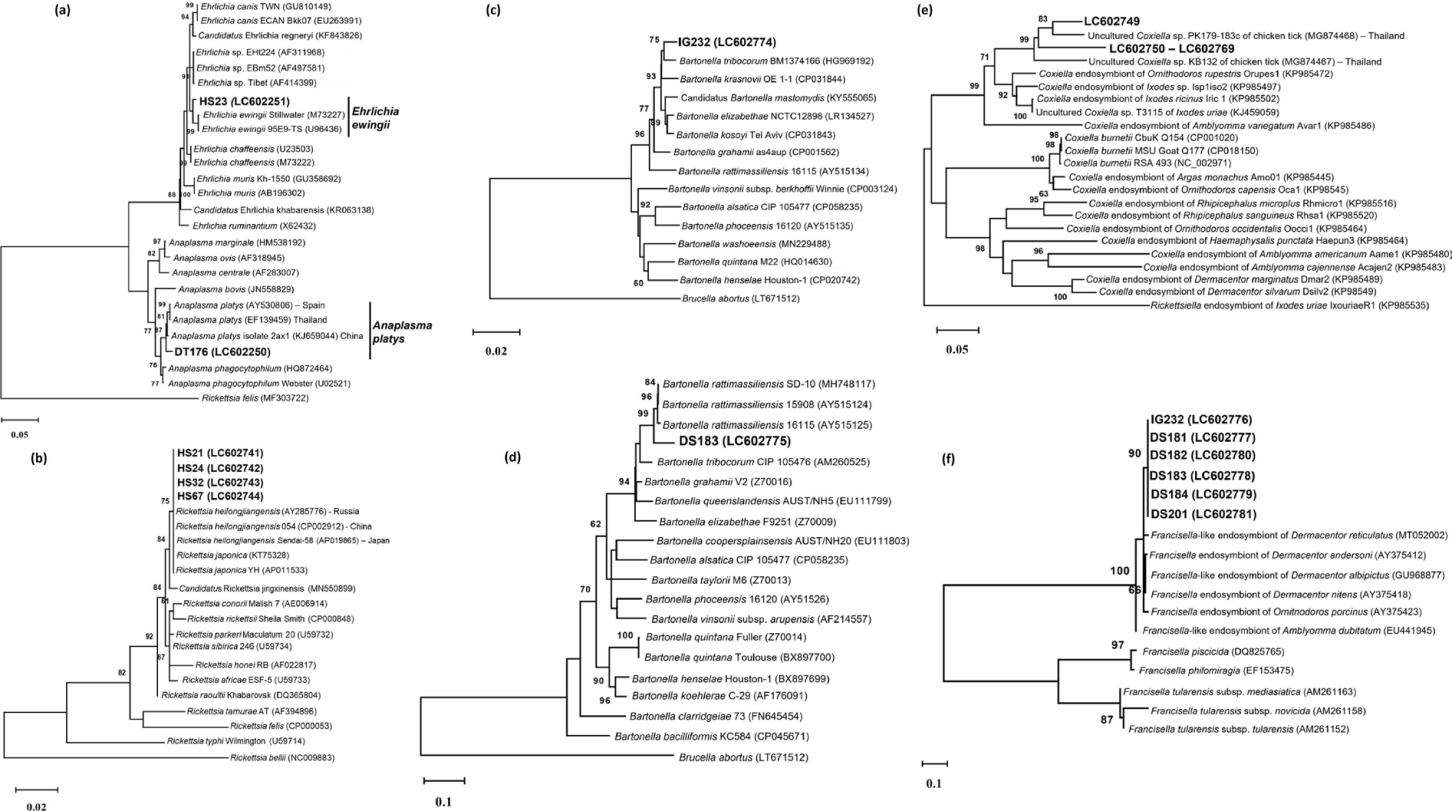
Phylogenetic trees of detected bacterial species based on the sequences of each respective gene used: (a) *Anaplasmatacea* (16S rDNA); (**b**) *Rickettsia* (*gltA* gene); (**c**) *Bartonella* (*ftsZ* gene); (**d**) *Bartonella* (*gltA* gene); (**e**) *Coxiella* (*groEL* gene); and (f) *Francisella* (*tul*4 gene). All trees are drawn to scale, with branch lengths measured in the number of substitutions per site. Samples and accession numbers from this study are in bold type. The analyses were performed using the neighbour joining or maximum likelihood methods. Bootstrap values >60 % based on 1000 replications are indicated on the interior branch nodes. More phylogenetic trees based on other genes used in bacteria species characterization are available (Fig. S5).

*Coxiella* sp. closely related to *Coxiella *-LE was identified in *H. shimoga* and *H. hystricis* ticks with 86.9–99.8% identity (Table 4; Figs 11 and S5). *Francisella* sp. closely related to *Francisella *-LE strain FLE011 from *Hyalomma marginatum marginatum* in Bulgaria (HQ705174) was identified from *D. steini* and *I. granulatus* ticks with 94.9 % (203/214 bp) identity based on the *tul4* gene (Table 4; Fig. 11). In addition, *Francisella* sp. closely related to a *Francisella* endosymbiont of *D. atrosignatus* isolate DASSD4 in Thailand (KC170748) was identified in *D. steini* with 99.9 %(1100/1101 bp) identity based on 16S rDNA (Table 4; Fig. S5). Furthermore, *Rickettsia heilongjiangensis,* the causative agent of spotted fever rickettsiosis in humans, was identified from *H. shimoga* ticks with 99.9–100% identity to *R. heilongjiangensis* Sendai-58 detected in *Haemaphysalis concinna* (AP019865) in Japan. The phylogenetic trees constructed using multiple target genes also showed the clustering of the detected *Rickettsia* with *R. heilongjiangensis* (Figs 11 and S5). We also detected *Anaplasma* sp. closely related to *A. platys* isolated in deer in China (KJ659044), with 98.9 % (1313/1327 bp) identity from *D. atrosignatus*. In this study, *Ehrlichia* sp. identified from *H. shimoga* had 98.9 % (1315/1330 bp) identity with the *Ehrlichia* sp. EBm52 of *Rhipicephalus microplus* reported in Thailand (AF497581). The phylogenetic tree based on 16S rDNA for *Anaplasma* and *Ehrlichi* a showed that the *Anaplasma* sp. and *Ehrlichia* sp. in this study clustered with *A. platys* and *E. ewingii *, respectively (Fig. 11). In addition, two *Bartonella* spp. were identified from *D. steini* and *I. granulatus* ticks. *Bartonella* sp. closely related to *B. rattimassiliensis* isolated from the European *Rattus norvegicus* in France (AY515124), with 97.8 % (703/719 bp) identity was identified in *D. steini* based on the *gltA* gene (Fig. 11). *Bartonella* sp. closely related to *B. tribocorum* strain MVT04 detected in human blood in France (HG969192), with 97.1 % (868/894 bp) identity was identified in *I. granulatus* ticks based on the *ftsZ* gene (Fig. 11).

## Discussion

Microbiome investigation in ticks has developed in the past decade due to the significance of tick-associated microorganisms for tick biological processes [55]. The interaction between the tick-associated microorganisms (pathogenic and non-pathogenic) has been positively or negatively associated with the mechanisms of colonization and transmission of pathogens. Recently, targeting the tick microbiome has been considered a potential approach in controlling ticks and tick-borne diseases. However, there has been no study on tick microbiomes reported from Sarawak, Malaysian Borneo, up to now. Furthermore, studies on tick species and TBDs have also been very limited. Here, we describe the microbial variations from six tick species of three genera: *Ixodes, Haemaphysalis*, and *Dermacentor* collected from primary forests and an oil palm plantation in Sarawak. We characterized the potential symbionts of each tick species, as well as the potential human and animal pathogenic bacteria detected from different tick species. Further, we explored the effects of *Borrelia* on the microbiota of *I. granulatus* ticks. Our study is the first initiative to outline different tick microbiome profiles in addition to screening of the pathogens from this region and included tick species that have never been studied previously.

In this study, comparative analysis with alpha and beta diversities revealed a significant difference in microbial diversity and composition in ticks. We found that the genus *Dermacentor* had higher microbial diversity, and *H. shimoga* had significant microbial composition differences with other tick species. A previous study on ticks from peninsular Malaysia did not detect any microbial diversity difference between *Dermacentor* (same *Dermacentor* species as in our study), *Amblyomma* and *Haemaphysalis* (*H. hystricis*) species collected from wild boar [32]. The difference with our study could be related to the blood meal feeding, as most of the *Dermacentor* ticks in our study were collected from vegetation. Some studies have reported an association between the blood meal host and tick microbiome [25, 56, 57] and implied that host blood is generally low in bacterial richness [56]. However, the possibility that the difference observed was due to the stochasticity of having a small sample size could not be excluded. While the blood meal explanation may be true for most tick species, including *I. granulatus* in this study, it did not explain the lower microbial diversity in *H. shimoga* (Fig. 2a). The result was also consistent when only adult ticks were included in the analysis, which consisted of only *H. shimoga* collected from vegetation (Fig. 2b). However, a high relative abundance of symbionts has been found to obscure detection of DNA sequences of the rare bacterial community in *Ixodes* ticks [24], which could explain the lower microbial diversity of *H. shimoga* in this study. Downstream analysis revealed that *Coxiella* dominated the microbiome in *H. shimoga* with as high as 80 % relative abundance in adult female ticks. Furthermore, the high abundance of *Coxiella* in *H. shimoga* might have resulted in the significant difference in microbial composition we observed in this study compared with other tick species (Fig. 3; Table S3).

Although there were variations in tick samples such as blood meal status, developmental stage and sex, we did not find any significant difference in alpha and beta diversity analyses. One of the reasons could be that most ticks were collected from vegetation, except for *I. granulatus* ticks which were all engorged, and some tick species did not have or had few nymph and larva samples (Table 1). To overcome the variation that could have resulted from ontogenetic differences, we compared between all ticks and only adult ticks (Fig. 1). For instance, we found no significant difference among adult *Dermacentor* species in microbial richness, whereas analysis with all tick samples showed a difference (Fig. 2; Table S2). In addition, adult *H. shimoga* ticks formed distinct clusters from other tick species in PCoA plots (Fig. 3b), showing that the adult *H. shimoga* ticks had significantly different microbial compositions. These findings supported that ontogenetic variation could contribute to the differences in the bacterial community in ticks. Ontogenetic variation in the tick microbiome has been reported in previous studies [27, 58]. For example, *Rickettsia* was determined as the main symbiont of *I. scapularis*, but the relative abundance reduced from larvae to adult ticks, except for adult females [27]. Conversely, Chicana *et al*. [58] reported the increased relative abundance of symbionts across developmental stages in most tick species they observed in their study.

We identified the potential symbionts in each tick species, except for *D. compactus* and *D. atrosignatus*. The main symbionts have frequently been reported in ticks, and some of their functional roles have been determined. In this study, the order *Rickettsiales* ws detected from all the examined tick species. Primarily, *Rickettsiales* dominated in *I. granulatu*s, *H. hystricis* and *D. steini* (Table 2). The *Rickettsiales* group consists largely of pathogenic agents under genera *Anaplasma, Ehrlichia, Rickettsia* and *Wolbachia* [59]. We were also able to characterize *Anaplasma* sp., *Ehrlichia* sp. and *R. heilongjiangensis *. However, the rest of *Rickettsiales* strains remained unidentified. *Rickettsia* symbionts have commonly been reported in arthropods and have also been reported in some tick species [21, 60, 61]. In other arthropod species, *Rickettsia* symbionts functioned as reproductive manipulators [18] and had defensive mechanisms in other insects [62], but their role in ticks is still not fully understood. With the high proportion of *Rickettsiales* detected in this study, it could be speculated that *Rickettsia* symbiont might be dominating in *I. granulatus, H. hystricis,* and *D. steini*, which requires further clarification.

In addition, we found *Candidatus* Rhabdochlamydia, intracellular bacteria of the order *Chlamydiales *, in high relative abundance in most of the tick species, including 2.57 % in *D. steini. Chlamydiae* DNA has been reported in *Ixodes ricinus* [63–65] and Australian ticks, such as *Ixodes tasmani*, *Ixodes holocyclus* and *Haemaphysalis humerosa* [66]. In addition, Pillonel *et al*. [64] reported *Rhabdochlamydia helvetica* as the obligate intracellular symbiont of *I. ricinus*. Although the species of *Candidatus* Rhabdochlamydia were not characterized in this study, it is important to know that most of the tick species were capable of harbouring the bacteria. This warrants future investigation as *Candidatus* Rhabdochlamydia spp., such as *C*. R. porcellionis [67] and *C*. R. crassificans [68] have been reported to be detrimental to their hosts.

*Francisella* has been reported from several tick species, and recently Duron *et al*. [17] demonstrated that this bacterium was maternally inherited and essential in haematophagous ticks for vitamin B synthesis. In this study, we characterized *Francisella *-LE from *D. steini* and *I. granulatus*, but not from *D. compactus* or *D. atrosignatus*. Furthermore, there were no potential symbionts identified from *D. compactus* or *D. atrosignatus*. Although our sample size for these two species was small, most of the bacterial taxa detected had less than 1 % relative abundance, with a few taxa exceptional, but also below 10 %. Adults of these three *Dermacentor* species feed chiefly on wild boars (Suidae), and a wide range of mammalian hosts, including humans and reptiles, have also been reported [69–71]. It is interesting that despite the similarity of the feeding hosts, the microbial composition was different from that of *D. steini*. A previous study suggested that ecological and physiological factors could play a role in shaping the tick microbiome and allow the tick not to require a dominant symbiont to survive and develop [58]. Our findings for *Dermacentor* also differed from a previous study in Malaysia [32] with their samples collected from wild boar. Therefore, investigating environmental and host association factors in these species would be of particular interest.

Potential symbionts were identified for *H. shimoga* with ontogenetic and sex variations. For instance, a study has demonstrated that *Coxiella *-LE is essential for *Amblyomma* tick survival and reproduction [72]. The organism is also commonly reported from many other tick species [73, 74]. In this study, we found *Coxiella *-LE (characterized by PCR) as the potential symbiont for *H. shimoga*, with an overall relative abundance of 50 % and was highest in female ticks (80, and 62 % in male ticks). *Coxiella *-LE was reported to provide vitamin B and co-factor essential for haematophagous arthropods such as ticks [75, 76]. So, its presence in adult ticks and nymphs (25.8 %) may be necessary for feeding and reproduction for female ticks. However, *Coxiella *-LE was only present at 8.09 % in larva samples, contrary to previous reports as a maternally inherited organism in *Amblyomma, Rhipicephalus* and *Ornithodoros* [77–80]. A recent study by Ben-Yosef *et al*. [81] has demonstrated that *Coxiella *-LE is essential for ontogeny development, fitness, fertility and fecundity in *Rhipicephalus sanguineus*, but also pointed out that it may not be mandatory for oocyte development and hatching. It is not clear for *H. shimoga* whether the other bacteria taxa have replaced the *Coxiella *-LE functional role in the larva. However, *Rickettsiales* and *Candidatus* Rhabdochlamydia were the potential symbiont observed from the larva samples. In addition, we could not rule out that ticks obtained *Coxiella *-LE through feeding, as previous studies have detected it from the salivary gland of some tick species [79, 80, 82]. Furthermore, tick-transmitted *Coxiella *-LE was responsible for mild infectious cases in humans [83], which means the organism could be transmitted via tick bites. It is noteworthy that human is a documented host for *H. shimoga* ticks we collected from the oil palm plantation [84]. Finally, while *Coxiella *-LE was the single potential symbiont in adult male and female ticks, *Coxiella *-LE and *Rickettsiales* were equally dominant in nymph samples and likewise for the larva samples. Overall, it was evident that ontogeny affected the microbial structures in *H. shimoga*, as different bacterial taxonomies were seen to dominate in different developmental stages, with *Coxiella *-LE present mainly in adult ticks.

In this study, we detected high proportion of *Borrelia* in *I. granulatus*, and the presence of *Borrelia* has a significant effect on tick microbial composition. A high proportion of *Borrelia* was reported in *I. scapularis*, in which the authors found microbial composition differences between *Borrelia *-positive males and females collected from two regions [85]. In their study, geography was significantly related to pathogen detection. Consistently, in this study, the majority of the *Borrelia *-positive *I. granulatus* ticks were collected from the oil palm plantation [12]. While environmental factors could have contributed to the high relative abundance of *Borrelia* in the ticks, other factors such as sex and blood feeding could not be examined in our study, as our *I. granulatus* samples were all engorged females. A previous study has revealed that *Ixodes ricinus* nymphs infected with *Borrelia afzelii* survived better than non-infected nymphs in a desiccating environment [86]. Furthermore, temperature changes could affect the tick life cycle and activity, which could have a profound effect on tick-borne pathogens. However, little is known about the impact of raised temperatures on the tick microbiota, as a long-term study is required to obtain such evidence [87]. Other than *Borrelia *, several other bacteria species such as *Anaplasma* sp., *Ehrlichia* sp., *Bartonella* spp., *R. heilongjiangensis,* and *Coxiella* and *Francisella *-LE were also identified from different tick species (Table 4; Figs 11 and S5). Other than *R. heilongjiangensis,* which causes spotted fever rickettsiosis in humans, *Anaplasma* sp. and *Ehrlichia* sp. in this study clustered with *A. platys* and *E. ewingii* in the phylogenetic tree, respectively. *Anaplasma platys* is known to cause canine cyclic thrombocytopaenia in dogs, while *E. ewingii* caused human monocytic ehrlichiosis in humans. However, due to the low number of samples and low detection rate, correlation analysis was not conducted. Nevertheless, given that there has been human serological evidence for *Anaplasma *, *Ehrlichia* and *Rickettsia* reported previously from Malaysia [8, 9], the identification of pathogenic and potentially pathogenic bacteria species in this study warrants more investigation. Furthermore, as the positive tick species such as *H. shimoga* and *Dermacentor* species have a reported wide range of feeding hosts, including humans, it is important to understand the actual prevalence and transmission mechanism. Besides, *Coxiella *-LE and *Francisella *-LE were closely related to their pathogenic type and shifting between pathogenic and non-pathogenic forms could occur. For example, *Coxiella burnetii *, the aetiological agent of Q fever, was clustered in the same clade with symbionts of soft ticks in the phylogenetic tree [73].

All in all, this is the first multispecies microbial comparison in ticks collected from Sarawak, Malaysian Borneo, with the identification of human and animal pathogens. Our findings revealed that microbial variations were significant between tick species. Potential factors contributing to the variations, including blood meal feeding and the presence of a single symbiont in high abundance, may have played a role in shaping the microbiome profile. Further investigation with *H. shimoga* revealed the ontogenetic and sex variations affecting microbial composition, with some bacterial taxa found to be more represented in one developmental stage than another. However, comparing the feeding status of *H. shimoga* did not show conclusive results (Tables S2 and S3; Fig. S1), probably because questing ticks consisted mainly of adults. Only nymph and larva samples were in the feeding category (Table 1). Most tick species in this study harboured one or multiple potential symbionts, except for *D. compactus* and *D. atrosignatus*. We speculated that other factors such as ecological variation could probably have a greater effect on *D. compactus* and *D. atrosignatus*, enabling them to survive and develop without a main symbiont, which requires further study. Finally, tick microbial structure could be affected by the presence of specific bacteria taxa in high abundance, as evidenced by *Borrelia *-positive and *Borrelia *-negative *I. granulatus*. This study’s biggest limitation is that the tick samples were collected with no prior data on tick species distribution in Sarawak and therefore yielded unequal sample numbers of each tick species. In addition, quantitative PCR provides an accurate estimation of the microbial burden, which has been used in other studies [24, 58, 81] and should be included in future studies, especially when comparing across developmental stages. Nevertheless, our study findings provide important insights into tick microbiome differences and the presence of pathogenic and potentially pathogenic bacteria circulating in ticks from primary forests and an oil palm plantation in Sarawak. More studies are required to unravel the potential functional roles of tick microbiomes observed in this study, particularly through isolation and *in vivo* inoculation of bacterial symbionts into ticks and monitoring the associated physiological changes using advanced proteomics and metabolomics.

## Supplementary Data

Supplementary material 1
